# How Long Does a Neutrophil Live?—The Effect of 24 h Whole Blood Storage on Neutrophil Functions in Pigs

**DOI:** 10.3390/biomedicines8080278

**Published:** 2020-08-08

**Authors:** Marta C. Bonilla, Leonie Fingerhut, Adriana Alfonso-Castro, AhmedElmontaser Mergani, Cornelia Schwennen, Maren von Köckritz-Blickwede, Nicole de Buhr

**Affiliations:** 1 Institute for Physiological Chemistry, University of Veterinary Medicine Hannover, Foundation, 30559 Hannover, Germany; Marta.Cristina.Bonilla.Gonzalez@tiho-hannover.de (M.C.B.); Leonie.Fingerhut@tiho-hannover.de (L.F.); a.alfonsocastro@ufl.edu (A.A.-C.); Ahmed.Mohamed@tiho-hannover.de (A.M.); Maren.von.Koeckritz-Blickwede@tiho-hannover.de (M.v.K.-B.); 2 Research Center for Emerging Infections and Zoonoses (RIZ), University of Veterinary Medicine Hannover, Foundation, 30559 Hannover, Germany; 3 Clinic for Horses, University of Veterinary Medicine Hannover, 30559 Hannover, Germany; 4 College of Veterinary Medicine, University of Florida, Gainesville, FL 32608, USA; 5 Clinic for Swine, Small Ruminants and Forensic Medicine and Ambulatory Service, University of Veterinary Medicine Hannover, 30173 Hannover, Germany; cornelia.schwennen@tiho-hannover.de

**Keywords:** neutrophils, life span, porcine blood, antimicrobial activity, reactive oxygen species (ROS), neutrophil extracellular trap (NET) formation, phagocytosis

## Abstract

Neutrophils are important effector cells of the innate immune system, traditionally regarded to have a short life span. The goal of this study was to evaluate the effect of the whole blood storage on neutrophil functions, e.g., viability, antimicrobial effect, neutrophil extracellular trap (NET) formation and phagocytosis. Therefore, fresh porcine whole blood was compared to whole blood stored for 24 h in the dark at room temperature. Different cell parameters in whole blood and in isolated neutrophils were analyzed. The following parameters were analyzed: cell count, band and segmented neutrophil count, viability, cholesterol content, release of free DNA as a marker for cell death, phagocytic activity in whole blood and in isolated neutrophils, the transmigration rate of neutrophils to IL8 stimulus, the production of reactive oxygen species (ROS), and the formation of NETs. It was observed that the number of isolated neutrophils decreased over time, indicating cell death occurs during 24 h of blood storage. However, the surviving neutrophils isolated from stored blood reacted comparably or even showed enhanced antimicrobial activity in the case of phagocytosis of *Streptococcus* (*S.*) *suis*, ROS production, and transmigration. The slightly altered cholesterol level of the harvested neutrophils in stored blood when compared to fresh blood partially explains some of the detected differences.

## 1. Introduction

Neutrophils are one of the most abundant leukocytes in blood circulation. In humans around 50–70% [1] and in pigs around 15–72% [2,3,4] circulating leukocytes are neutrophils. Furthermore, they are one of the first cells infiltrating into infected tissue [1]. Neutrophils have different intra- and extra-cellular mechanisms to eliminate microbes [5]. One intracellular mechanism is phagocytosis where neutrophils engulf the microbe in the phagosomal machinery and kill the microbe with different antimicrobial components, for example, myeloperoxidase, neutrophil elastase or antimicrobial peptides [6,7]. These different components can kill a wide variety of microorganisms. Furthermore, in the phagosome, the production of reactive oxygen species (ROS) is initiated and a decrease in pH occurs, leading to a powerful antimicrobial activity [6]. Furthermore, neutrophils can release these antimicrobial components from vesicles/granules to the extracellular space. This process is called degranulation of the neutrophil [5]. Another extracellular mechanism for controlling pathogens is the release of neutrophil extracellular traps (NETs) [8]. In this mechanism, neutrophils release DNA fibers decorated with antimicrobial molecules. Pathogens can be killed or at least immobilized inside these NETs and therefore the pathogen spreading is prevented [5,6]. Despite being such important cells and having different functions in the control of invading microorganisms, neutrophils have been reported to be short-living cells. There are reports that neutrophils have a circulating half-life of less than 8 h in humans [9,10,11]. When used ex vivo in culture, the half-life of the neutrophil was shown to be less than 24 h [12]. A single study reported that neutrophils could have a half-life of around 5 days [12]. However, this brought some controversy [13] and finally a contradictory study was conducted that disproved the theory of a half-life of approximately 5 days. It was again described that neutrophils have a half-life of less than 24 h [14].

Given neutrophils’ important role in immune response and their use in research, it is necessary to understand their viability and function ex vivo. When working with fresh blood to isolate neutrophils, how long the blood can be stored for before the isolation procedure begins is a topic of continuous debate. In the case of animal experiments or clinical studies, it is not always possible to directly harvest neutrophils from blood of animals or humans due to technical reasons. For this reason, the aim of this study was to investigate neutrophil viability and antimicrobial activity after being stored in whole blood for 24 h at room temperature when compared to fresh blood. This time-point was furthermore chosen as this would allow an over-night express shipping of blood to the respective lab.

## 2. Results

### 2.1. Analysis of Cell Parameters in Fresh and 24 h Stored Whole Blood from Pigs

**Figure 1:**
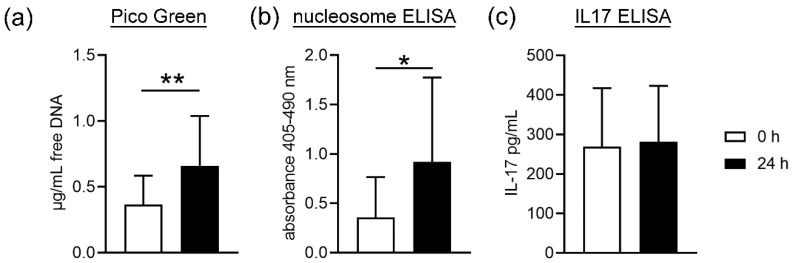
(**a**) Significantly higher concentrations of free DNA and (**b**) nucleosome were detected in the plasma obtained from stored blood. (**c**) The amount of IL17 was not different in plasma from fresh and stored blood. Data were analyzed with one-tailed paired Student’s *t*-test (a-b: *n* = 10; c: *n* = 9) and presented with mean ± SD, (* *p <* 0.05, ** *p <* 0.01).

To assess whether cell death in stored blood is significantly altered, we analyzed the concentration of free DNA and nucleosome in plasma as a marker for cell death [15]. Furthermore, IL17 was determined as a cytokine that is involved the recruitment of neutrophils [16] and neutrophils can produce IL17 on their own to enhance the recruitment of more cells [17]. Interestingly, it was shown that IL17 could enhance NET release and is associated with NETs [16,18]. For this reason, the measurement of the amount of IL17 in combination with free DNA and nucleosome is a tool to quantify NET-associated cell death in serum and plasma [18,19]. The plasma obtained from stored blood contained significantly higher concentrations of free DNA (*p =* 0.0075) and nucleosome (histone-complexed DNA fragments) (*p =* 0.0264), suggesting increased cell death (Figure 1a,b). ELISA-based quantification showed no significant difference in the amount of IL17 plasma obtained from fresh or stored blood (Figure 1c). Nevertheless, as IL17 is described to be bound to NETs only under some circumstances, these results only suggest the possibility that the detected increase in cell death is not NET dependent. Further analysis is needed to clarify this.

**Figure 2:**
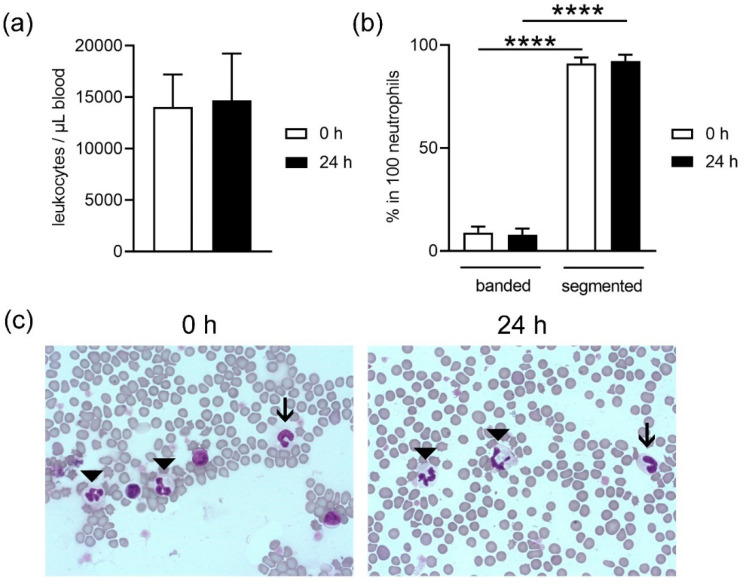
(**a**) No significant difference in the number of leukocytes was detected in whole blood. (**b**) Amount of band and segmented neutrophils in stained blood smears. (**c**) Representative images show the morphology of neutrophils in fresh (0 h) and stored blood (24 h) after HAEMA fast stain (band = arrow, segmented = arrowhead). The images were captured with a Zeiss Axio Imager M2 microscope and taken with a 63× objective. Data in a and b were analyzed with one-tailed paired Student’s *t*-test (*n* = 9) and presented with mean ± SD (**** *p <* 0.0001).

As neutrophils are described and discussed as short-living cells [14], we were interested if differences in the neutrophil population can be identified after storage. Because they belong to the leukocyte population, we analyzed first the total number of leukocytes. However, a total leukocyte count showed no significant difference between fresh and stored blood (Figure 2a). The leukocyte count (mean 0 h = 14.067 ± 3.147 leukocytes/μL, mean 24 h = 14.694 ± 4.54 leukocytes/μL) aligned with reference values for pigs (11.000–22.000 leukocytes/μL [20]). Furthermore, as a sign of neutrophil maturation and aging, we analyzed the amount of band (young) and segmented (old) neutrophils in stained blood smears (Figure 2b,c). Independent of the storage, more segmented neutrophils than bands were detected in the blood smears. A counting of 100 neutrophils in each sample resulted in 91.1 ± 2.89% (0 h) and 92.2 ± 3.11% (24 h) segmented neutrophils and only 8.8 ± 2.89% (0 h) and 7.7 ± 3.11% (24 h) band neutrophils (Figure 2b,c). These data show that there is no change in the leukocyte population of stored blood when compared to fresh blood.

### 2.2. Analysis of Cell Parameters in Neutrophils Isolated from Fresh and 24 h Stored Blood from Pigs

**Figure 3:**
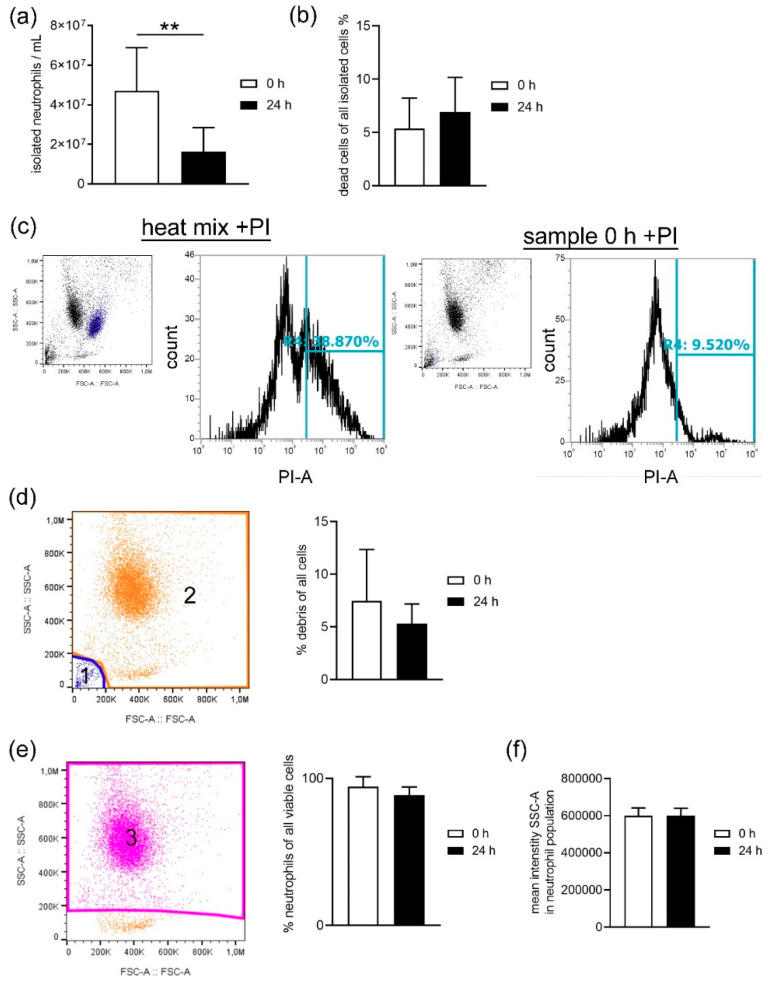
(**a**) Significantly less neutrophils (counted in a Neubauer chamber) were isolated from stored blood. (**b**) Slightly but not significant higher percentage of dead cells were detected in isolated cells from stored blood. (**c**) The gating strategy for the dead cell analysis after PI (propidium iodide) staining by flow cytometry is presented. Settings were adjusted with a 1:2 mix of heat-killed and living cells (heat mix). Blue points in the dot blot depict the dead cells identified by the histogram and respective gating (left panel). (**d**,**e**) The isolated cells were analyzed by flow cytometry for purity and granularity. (**d**) Based on FSC-A (forward scatter area) and SSC-A (side scatter area), the debris (blue dots) were excluded and viable cells were marked orange. The amount of debris was not significant different between fresh and stored blood. (**e**) Based on FSC-A and SSC-A, the neutrophil population (pink dots) were gated in the viable cell population. No significant difference in the percentage of neutrophils in the viable cell population was found. (**f**) The mean intensity SSC-A (granularity) was not different in the gated neutrophil population when compared with isolated cells from fresh and stored blood. Data were analyzed with one-tailed paired Student’s *t*-test (** *p <* 0.01) and presented with mean ± SD, (a: *n* = 7, b: *n* = 5, d-e: *n* = 4).

In the next step, we were interested in analyzing the number and viability of neutrophils isolated from fresh and stored blood. Significantly less neutrophils (*p =* 0.002) were isolated from stored blood (Figure 3a). We analyzed the percentage of dead cells after propidium iodide staining by flow cytometry and found a slightly higher percentage of dead cells in neutrophils isolated from stored blood (mean 24 h = 6.93 ± 2.86%) compared to fresh blood (mean 0 h = 5.36 ± 3.25%) (Figure 3b,c). These results suggest an increased cell death during the isolation of neutrophils from stored blood or a change in morphology consequently influencing the isolation process by density gradient. A flow cytometry analysis of the isolated cells showed no significant difference in the amount of debris (Figure 3d). Furthermore, the percentage of neutrophils in the population of viable cells was independent of the storage high (purity 0 h = 94.4%, purity 24 h = 88.24; Figure 3e) and the granularity was in the same range (Figure 3f). These results show that the isolated population of neutrophils do not change significant in their morphology. As the number of isolated and living neutrophils was high from the 24 h stored blood, we questioned whether the activity may differ between neutrophils isolated from fresh and 24 h stored blood.

### 2.3. Analysis of Transmigration in Fresh and 24 h Stored Whole Blood from Pigs

**Figure 4:**
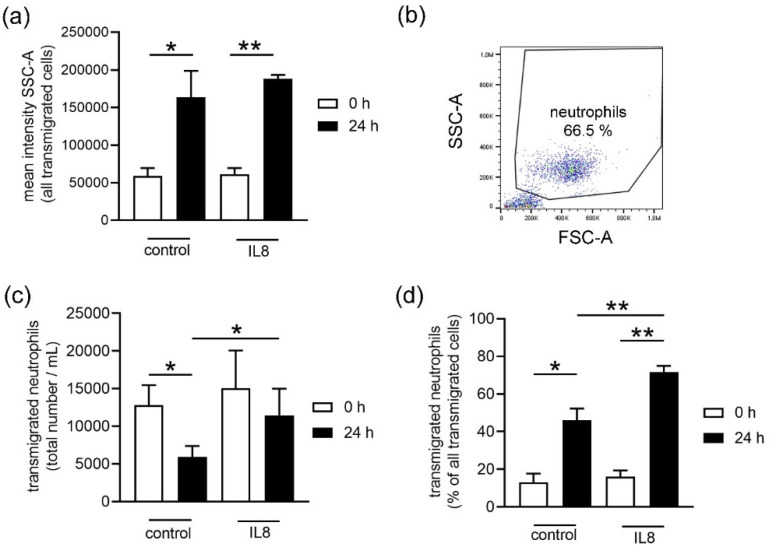
Neutrophil transmigration from whole blood was analyzed after 4 h incubation. (**a**) The mean intensity SSC-A (granularity) of all transmigrated cells was significant different between fresh and stored blood with and without IL8 treatment. It revealed higher granularity of transmigrated cells, indicating higher activation of neutrophils after 24 h of blood storage as also confirmed by ROS production, shown in Figure 5. (**b**) The gating strategy for the analysis of transmigrated cells by flow cytometry is presented and neutrophils were identified based on FSC-A and SSC-A. (**c**) Neutrophils from stored blood transmigrated less than neutrophils from fresh blood. The IL8 stimulated transmigration was only significantly increased in stored blood. (**d**) Observing the whole transmigrated cellular population, the percentage of neutrophils is significantly higher in stored blood. Data were analyzed with one-tailed paired Student’s *t*-test (all assays were conducted in triplicates, and the mean is presented from *n* = 3 independent runs) and presented with mean ± SD (* *p <* 0.05, ** *p <* 0.01).

Neutrophils can be attracted by chemokines and they can transmigrate out of the blood to the site of infection. One chemokine attracting neutrophils to side of infection is IL8 [21,22]. Therefore, the transmigration capacity of cells from whole blood to IL8 was analyzed as one cell activity parameter. Based on the analysis of mean intensity, the granularity of all transmigrated cells was analyzed by SSC-A (side scatter area) with flow cytometry. A significantly higher mean intensity was found in stored blood (Figure 4a). Afterwards, the transmigrated cells were gated based on FSC-A (forward scatter area) and SSC-A (Figure 4b). The total number of transmigrated neutrophils was significantly lower (*p* = 0.024) in IL8-untreated control cells in fresh (0 h = 12.818 ± 2.660 neutrophils/mL) and stored blood (24 h = 5.928 ± 1.468 neutrophils/mL) when compared to IL8-treated groups (Figure 4c). Independent of IL8 treatment, the total number of transmigrated neutrophils was generally lower in stored blood. These data align with the data showing that less neutrophils can be harvested from stored blood. Thus, since less neutrophils are present in stored blood, less neutrophils can transmigrate. However, to detect if the efficiency of the viable neutrophils is altered after blood storage, the percentage of transmigrated neutrophils among all transmigrated cells was calculated (Figure 4d). Importantly, compared to all transmigrated cells, significantly more neutrophils transmigrate from stored blood (24 h), regardless of whether cells were untreated (control, *p =* 0.0161) or IL8 treated (*p =* 0.0014). Furthermore, an IL8 specific transmigration of neutrophils was significantly higher in stored blood (Figure 4d, *p =* 0.0022). These data indicate that neutrophils from stored blood are more activated for transmigration as a response to IL8 stimulation when compared with neutrophils from fresh blood.

As the transmigration of neutrophils was influenced by storage, we wondered if maybe other activity parameters in the neutrophil population differ between neutrophils isolated from fresh and stored blood.

### 2.4. Analysis of ROS Activity and NET Formation in Neutrophils Isolated from Fresh and 24 h Stored Blood from Pigs

**Figure 5:**
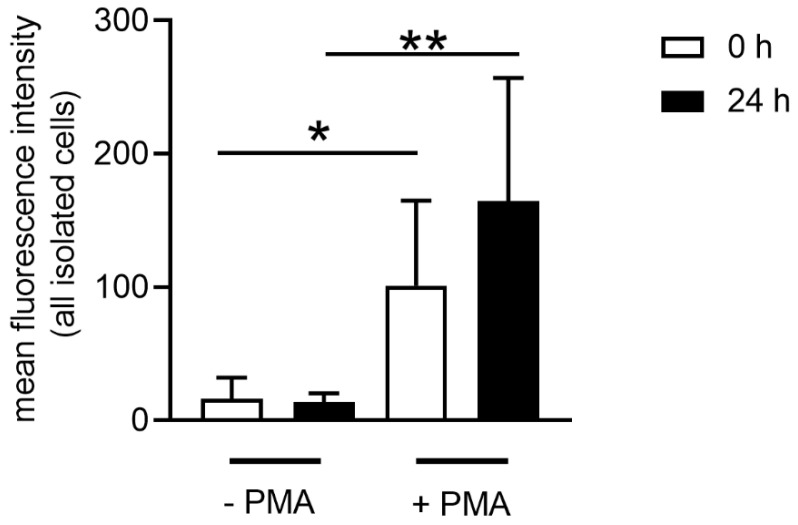
Isolated neutrophils from stored blood produced higher amounts of intracellular reactive oxygen species (ROS) after phorbol 12-myristate 13-acetate (PMA) stimulation. The intracellular ROS production was determined by adding 2′7′ dichlorofluorescein diacetate (DCFH-DA) to unstimulated and PMA-stimulated cells. Oxidation of DCFH-DA by ROS leads to fluorescence 2′,7′- dichlorofluorosceindiacetate (DCF). The fluorescence-positive cells were quantified by flow cytometer. Data were analyzed with one-tailed unpaired Student’s *t*-test (*n* = 3 experiments with duplicates are presented). Data are presented with mean ± SD (* *p <* 0.05, ** *p <* 0.01).

Reactive oxygen species (ROS) are major part of the innate immune defense and one of the key effectors in mediating antimicrobial activity of neutrophils. Activated neutrophils produce ROS at the site of infection, which can result, for example, in increased transmigration [23] or also enhance neutrophil extracellular trap (NET) formation and NET-mediated antimicrobial activity [24,25]. Therefore, as a first activity parameter in the neutrophil population, intracellular ROS production was measured by adding 2′7′ dichlorofluorescein diacetate (DCFH-DA) and flow cytometry measurement of the fluorescence signal [26]. Neutrophils isolated from fresh and stored blood released ROS after phorbol 12-myristate 13-acetate (PMA) stimulation. Compared to an unstimulated sample, the neutrophils from stored blood released significantly more ROS (24 h *p =* 0.0033), as did neutrophils from fresh blood (0 h *p =* 0.0101) (Figure 5). Although neutrophils released ROS after external stimulation independent of the storage conditions of blood, ROS release as response to PMA occurred to a lesser extent in neutrophils from fresh blood (Figure 5). This result confirms the detected higher granularity of transmigrated cells after 24 h of blood storage, as it indicates a higher activation of neutrophils after 24 h of blood storage (Figure 4).

**Figure 6:**
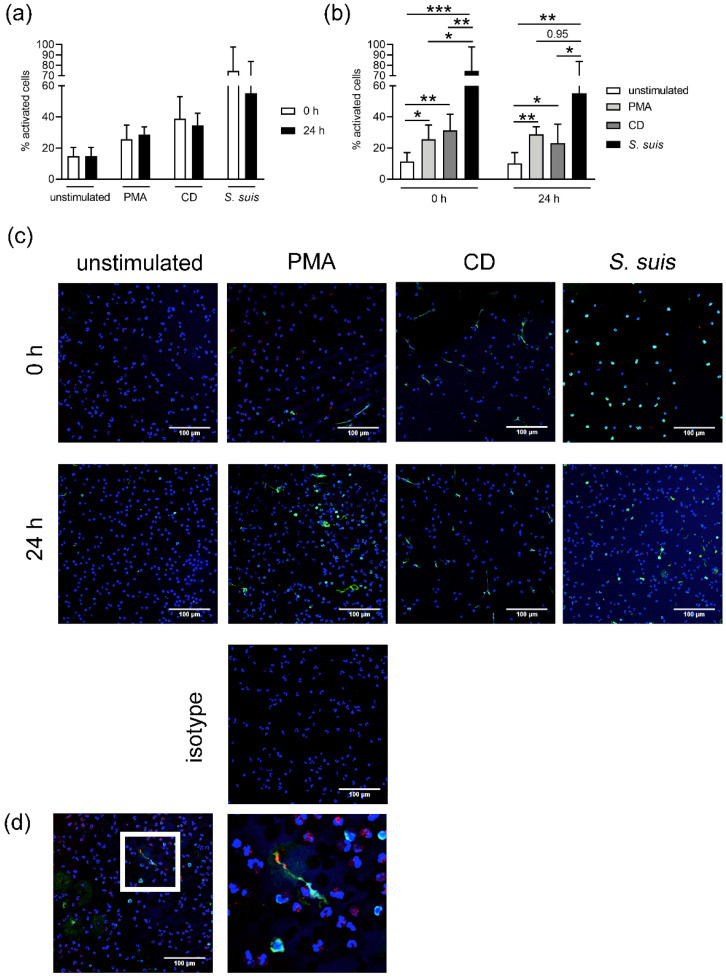
Neutrophil extracellular trap (NET) release was detected in neutrophils isolated from fresh and stored blood, independent of the stimulus. In each experiment and for each sample, six randomly taken pictures from two individual slides were analyzed for quantification. All cells on the six pictures were counted and the mean of activated cells per experiment was calculated and used for statistic. RPMI was used as unstimulated control. Methyl-β-cyclodextrin (CD) and phorbol 12-myristate 13-acetate (PMA) diluted in RPMI were used as positive controls. (**a**) Data are presented sorted by the stimulus. No significant difference was detected when comparing the different stimulations. (**b**) Data are presented sorted by the storage and statistic was calculated inside the respective storage group. Slightly higher NET release was detected in neutrophils from fresh blood after CD or *Streptococcus suis* (=*S. suis cps* type 2 strain 10) stimulation. Slightly higher NET release in neutrophils from stored blood was detected after PMA stimulation. Data were analyzed with one-tailed paired Student’s *t*-test (*n* = 3 PMA, *S. suis*; *n* = 5 CD, unstimulated) and presented with mean ± SD (* *p <* 0.05, ** *p <* 0.01, *** *p <* 0.001). In c and d, blue staining represents DNA (Hoechst), green marks DNA/histone-1-complexes and red denotes myeloperoxidase. (**c**) Representative images (overlay) of NET-induction assays used for quantification of activated cells are presented. The respective isotype control (PMA stimulated) used for the microscope settings is shown. (**d**) Shows a myeloperoxidase and DNA/histone-1 positive NET fiber. A triple zoom from the area marked with the white square is presented in the right image.

Based on the results from Figure 4 and Figure 5, we hypothesized that storing blood has an impact on the activity of neutrophils in the presence of bacteria. It has been described that NET formation is influenced by ROS production. The formation of NETs after stimulation with well-known chemical NET inducers (PMA and methyl-β-cyclodextrin = CD) and *Streptococcus (S.) suis* as a natural NET inducer was analyzed. While PMA triggers ROS-dependent NET formation, CD triggers a ROS-independent NET formation by the depletion of cholesterol from neutrophil membranes. Neutrophils from stored blood were able to release NETs independent of the stimulus (Figure 6). As shown in Figure 6a, neutrophils isolated from 0 and 24 h blood release in a similar amount NETs with the same stimulus. Nevertheless, if the NET release is compared only in the group of fresh and stored blood, small differences are found (Figure 6b). The neutrophils from fresh blood showed the strongest reaction after incubation with *S. suis cps* type 2 strain 10 (74.52 ± 23.2%), followed by CD causing the second strongest reaction (31.23 ± 10.49%) in this group. The stimulus that caused the lowest amount of NET release among neutrophils isolated from fresh blood was PMA (25.71 ± 9.01%). The neutrophils from stored blood also showed the strongest reaction after incubation with *S. suis cps* type 2 strain 10 (55.26 ± 28.6%). PMA, however, caused the second strongest reaction among this group (28.81 ± 4.78%). The stimulus that caused the lowest amount of NET release by neutrophils from stored blood was CD (23.2 ± 12.18%). Concurring with their higher ROS production (Figure 5), neutrophils from stored blood release more NETs after PMA stimulation than neutrophils from fresh blood. However, after CD stimulation, less NETs were released by neutrophils isolated from stored blood as compared to neutrophils isolated from fresh blood.

### 2.5. Analysis of Cholesterol Content in Neutrophils Isolated from Fresh and 24 h Stored Blood from Pigs

It is known that a decreased level of cholesterol leads to an increased spontaneous NET release [27]. Furthermore, cholesterol modulates NADPH (nicotinamide adenine dinucleotide phosphate oxidase) oxidase activity [28,29]. Cholesterol in general is described to be involved in the cell homeostasis, cell death, cytokine release and the host-pathogen interaction in various cell types [30,31]. Therefore, in the next step, the cholesterol content in neutrophils from stored blood compared to fresh blood was analyzed.

**Figure 7:**
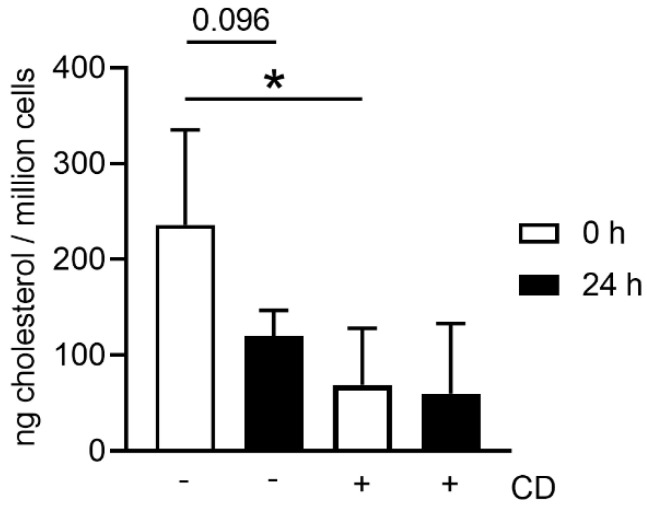
Neutrophils isolated from fresh blood contain more cholesterol than neutrophils isolated from stored blood. Methyl-β-cyclodextrin (CD) was used as control to reduce cholesterol content in the cells. A significant decrease in cholesterol after CD treatment was only detectable in neutrophils from fresh blood. Data were analyzed with one-tailed paired Student’s *t*-test (*n* = 3) and presented as mean ± SD (* *p <* 0.05).

A remarkable higher amount of cholesterol was detected in neutrophils from fresh blood compared to neutrophils from stored blood (0 h = 236.17 ± 99.03 ng cholesterol/million cells; 24 h = 120 ± 26.76 ng cholesterol/million cells). As a control experiment, a treatment with the cholesterol depleting agent methyl-β-cyclodextrin (CD) significantly decreased the cholesterol in neutrophils from fresh blood (*p =* 0.0315) (Figure 7). As already observed, a low cholesterol content was detected in the unstimulated neutrophils from stored blood; the depletion of cholesterol by CD was not significant in this group.

### 2.6. Analysis of Antimicrobial Activity of Fresh and 24 h Stored Whole Blood and Isolated Neutrophils from Fresh and 24 h Stored Blood from Pigs

**Figure 8:**
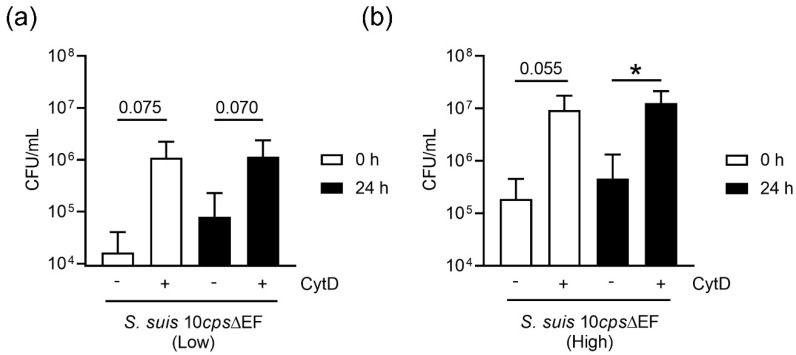
Storing the blood did not significantly influence the phagocytic activity of cells in whole blood. Phagocytosis was blocked by adding cytochalasin D (CytD). Two different initial infection doses were used: (**a**) A low infection dose = 1.5 × 10^5^ CFU/mL and (**b**) a high infection dose = 1.5 × 10^6^ CFU/mL). Data were analyzed with one-tailed paired Student’s *t*-test (*n* = 4) and are presented with mean ± SD (* *p* < 0.05). The *y*-axis is formatted to log 10.

Finally, we analyzed the antimicrobial activity in whole blood and of isolated neutrophils against the capsule mutant of *S. suis* as a model bacterium. This mutant strain without a capsule was used as it is sensitive to phagocytosis [32]. First, we analyzed the phagocytic activity in whole blood. As shown in Figure 8, immune cells present in porcine whole blood were able to phagocytize the capsule mutant of *S. suis* at comparable values independent of the infection dose and storage. In the absence of cytochalasin D to block phagocytosis, the total colony forming unit (CFU)/mL was slightly higher in stored blood compared to fresh blood, but no significant difference was detected. By adding cytochalasin D to inhibit the phagocytosis, distinctly more bacteria were recovered from the wells of all groups, meaning that phagocytosis takes place in fresh as well as stored blood.

As a next step, we focused on isolated neutrophils and examined whether the same antimicrobial activity tendency was detected as also shown for the whole blood assay (Figure 8). It was noted that isolated neutrophils could phagocytize bacteria independent of blood storage. There is generally very little antimicrobial activity of the neutrophils when no plasma is present in the assays. However, in the absence of plasma but presence of cytochalasin D, there was a significant difference detectable in surviving bacteria when comparing neutrophils derived from fresh blood to stored blood. Under these chosen conditions, neutrophils from fresh blood were able to kill bacteria more efficiently. This aligns well with the higher percentage of NET-releasing cells among neutrophils from fresh blood after *S. suis* infection (Figure 6). In the presence of autologous plasma, there is in general a higher antimicrobial activity detectable in both groups, which is blocked in the presence of cytochalasin D. Furthermore, in the presence of autologous plasma, it was observed that neutrophils isolated from stored blood tended to kill more bacteria compared to neutrophils from fresh blood. This phenomenon aligns well with higher ROS activity in the same group (Figure 5), but it was not statistically significant.

**Figure 9:**
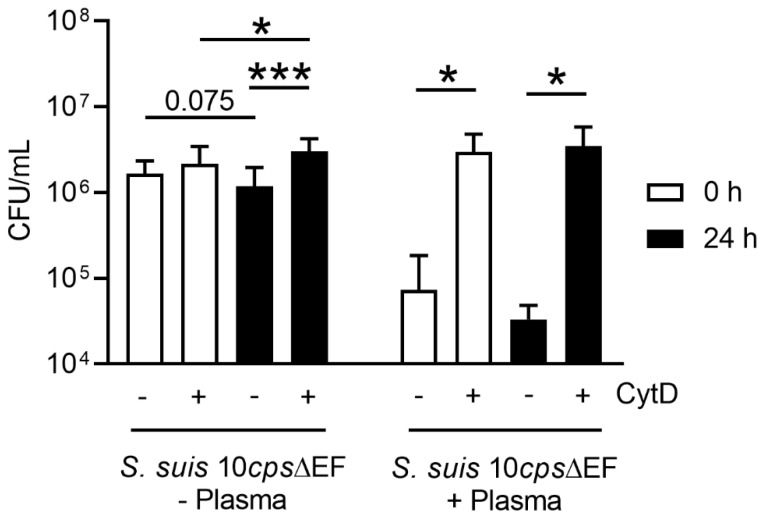
Neutrophils can phagocytize the bacteria independent of the storage. Slightly higher elimination of *S. suis* occurs with neutrophils from fresh blood when phagocytosis is inhibited. Slightly higher elimination of *S. suis* with neutrophils from stored blood occurs when phagocytosis is not inhibited. Data were analyzed with one-tailed paired Student’s *t*-test (*n* = 8, no autologous plasma) (*n* = 5, autologous plasma added) and presented with mean ± SD (* *p <* 0.05, *** *p <* 0.001).

In summary, Figure 8 and Figure 9 show that whole blood as well as isolated neutrophils from stored blood still shows efficient antimicrobial effect against *S. suis* capsule mutant.

## 3. Discussion

The differentiation and maturation of neutrophils are complex processes which take around 14 days in the bone marrow [33]. Despite this, neutrophils have a very short life-span in blood circulation, estimated to be less than 24 h [14]. However, this topic is controversially discussed, and some authors describe long-living neutrophils [11,12]. Thus, it is conceivable that blood cells die during storage of whole blood when they are planned to be used for ex vivo experiments. In clinical studies, it is of interest to work with material from patients, but an immediate processing of blood samples is not always possible, and time-consuming shipping/transport may be needed. Therefore, it is of interest to investigate if storing whole blood for 24 h has an impact on neutrophil harvest, viability, and activity.

First, cell death of blood cells was analyzed by studying free DNA and nucleosome in plasma. NET formation is one of the proinflammatory death pathways of neutrophils that involves cell membrane lysis and releases free DNA [34]. In this mechanism, the neutrophil releases a DNA backbone decorated with antimicrobial components [5,6]. The quantification of free DNA is a marker used to detect and quantify NETs [35]. Nevertheless, free DNA and nucleosome are not exclusively NET markers, as necrosis also result in free DNA. As shown in Figure 1, we found a significant increase in free DNA and nucleosome in the plasma samples from stored blood compared to fresh blood. This could be due to a higher cell death or membrane lysis from all blood cells, including neutrophils. As IL17 is next to other markers such as MPO or neutrophil elastase, only one NET marker [18] and did not differ in plasma from fresh and stored blood; thus, it may be hypothesized that the higher amount of free DNA does not originate from NET-releasing cells. Further analysis is needed to confirm this hypothesis. These data are confirmed by counting neutrophils after density gradient-based separation and harvest of neutrophils from fresh compared to stored blood. Slightly less neutrophils are harvested from stored blood compared to fresh blood, indicating cell death during storage time or isolation. In contrast, no significant difference between fresh and stored blood was detected for white blood cell counts (WBC) (Figure 2a), but the analysis did not differentiate between living and dead.

Neutrophils originate in the bone marrow from hematopoietic stem cells. Here, differentiation of cells occurs until the first progenitor of neutrophils is achieved, the promyelocyte. In the next stages, the cells develop granules, differentiation continues and the stage of band neutrophils is reached. These mature further and develop a segmented nucleus. This is the reason why they are also called polymorphonuclear granulocytes. Segmented neutrophils are the final stage of maturity [6,33,36]. Most of the neutrophils circulating in blood are mature neutrophils (segmented), but also band neutrophils can be found in a lower amount [1,2,3,4]. It is possible to find higher amounts of immature neutrophils circulating in blood due to acute infection or inflammation, where cells are needed quickly and, therefore, there is no time to finish maturation in the bone marrow [36]. In this study we used healthy blood donors and, as such, we expected to find a higher number of segmented neutrophils (Figure 2b,c). No differences were found in maturation of the band neutrophils between fresh or stored blood. Since the ratio did not shift, it can be speculated that segmented neutrophils do not exclusively die during storage.

When harvesting neutrophils from fresh or stored blood by density gradient centrifugation, the number of dead cells was slightly higher in neutrophils isolated from stored blood (Figure 3b), confirming the increased free DNA level shown for whole blood in Figure 1. Even though the number of leukocytes counted in whole blood by Leuko-TIC was roughly equal (Figure 2a), it seems that neutrophils from stored blood are more fragile. Therefore, it could be hypothesized that the steps during neutrophil isolation (e.g., the erythrocyte lysis step) do destroy more neutrophils from stored blood. One explanation for cells being more fragile is the lower content of cholesterol. Cholesterol is part of the neutrophil cell membrane and is involved in the process of adhesion and transmigration [37]. Our findings of lower cholesterol content in neutrophils isolated from stored blood (Figure 7) matches the hypothesis that neutrophils become more fragile and sensitive to rupture after storage. This would explain the significant higher number of isolated neutrophils from fresh blood compared to stored blood (Figure 3a). Furthermore, this aligns with the finding of more free DNA in the plasma of stored blood. An explanation could be that either the storage does not allow the cells to be preserved or their life time has ended, as previously described [14]. For this reason, it would be interesting to evaluate under other storage conditions, such as another temperature or storage time, if the number of neutrophils that can be isolated could be higher.

Despite this discussion, it is important to note that the amount that can be achieved from stored blood is enough for experiments to study neutrophil functions ex vivo. Therefore, we were interested to find out if these remaining neutrophils have normal activity and still could be used for ex vivo investigations.

Initially, when an infection or inflammation occurs in the body, neutrophils are recruited by chemoattractant molecules. One well described example is IL8: this chemokine can activate neutrophils, attracts them to the site of infection, and promotes their adhesion to the endothelium to finally transmigrate into the infected tissue [5,6]. Interestingly, the surviving neutrophils showed a higher transmigration rate in response to IL8 in stored blood compared to fresh blood (Figure 4b). Only neutrophils from stored blood transmigrated significantly more to the IL8 stimulus when compared to the unstimulated control group (Figure 4b). This suggests that storage could either increase a more specific reaction to stimulation or decrease the transmigration of other cells from stored blood or a combination of both.

Some metabolic reactions in cells produce reactive oxygen species (ROS), which are reactive molecules and free radicals derived from oxygen [38]. It is known that once neutrophils are activated or stimulated, they are able to produce ROS as molecules with antimicrobial potential [6]. When ROS is in contact with the pathogen it can cause damage in the cell membrane, nucleic acid and proteins [6]. These molecules can also be involved in cell activation and can enhance killing processes in neutrophils. For example, after neutrophils are stimulated with PMA, IL8 or a pathogen, the NADPH oxidase (nicotinamide adenine dinucleotide phosphate oxidase) complex in the neutrophil is activated and produces ROS. This enhances the reaction in the granules, combining the neutrophil elastase and myeloperoxidase with the chromatin, and finally they are released as NETs [15,24,39]. As ROS can be found in higher amounts when neutrophils are activated, we used ROS as a parameter for neutrophil activity. Indeed, ROS production was found in neutrophils from fresh and stored blood. This demonstrates that neutrophils are still active despite prior storage of blood (Figure 5). However, when neutrophils from stored blood are stimulated, they have slightly higher ROS activity compared to neutrophils derived from fresh blood. This again could be related to the cholesterol level of the cell since ROS-producing NADPH oxidase is found anchored in cholesterol-rich microdomains. In good correlation with our data, it has been reported that a reduced cholesterol content of the cell may lead to a higher enzymatic activity of the NADPH oxidase [40]. Similarly, we here show that neutrophils derived from stored blood show a decreased cholesterol level and at the same time increased ROS production.

As previously described, neutrophils can be stimulated to release NETs in the presence of pathogens, PMA, IL8, CD and others by different pathways [15,24,27,39,41]. We confirmed that both neutrophils from fresh and stored blood release NETs after incubation with *S. suis*, PMA or CD. However, the level of NET release varied depending on the stimulus (Figure 6). When neutrophils are stimulated with CD, NET release can occur in a short period of time (around 30 min) [27,41]. We identified the tendency of slightly higher NET release in neutrophils from fresh blood after stimulation with CD, which depletes cholesterol. In contrast with cholesterol, which mediates ROS-independent NET formation, PMA can induce NETs by activating the NADPH oxidase pathway and ROS production [15,39]. Neutrophils from fresh and stored blood release NETs and produce ROS after PMA stimulation. However, a slightly higher ROS production and NET release was observed in the neutrophils from stored blood, which aligns with higher ROS activity of those cells (Figure 5 and Figure 6).

Since neutrophils were isolated from porcine blood, *S*. *suis* was used as a natural stimulus for NET release. This pathogen is described as a zoonotic agent causing meningitis in pigs and it has been seen that neutrophils release NETs upon infection of this pathogen [42]. A higher release of NETs was found in neutrophils from fresh blood after stimulation with *S. suis* (Figure 6). This correlates with the data obtained in the neutrophil killing assay, especially when plasma is absent (Figure 9). Neutrophils from fresh and stored blood still have efficient phagocytic activity. It is well known [43] that neutrophils considerably improve pathogen elimination due to opsonizing antibodies or complements present in plasma and/or serum. Nevertheless, the overall phenotype might completely depend on the pathogen that is used for experiments. It could be possible that this tendency may change depending on the pathogen. As an example, NETs are known to act antimicrobial against *S. suis*, but in contrast pathogens such as *Actinobacillus (A.) pleuropneumoniae* have been reported to induce NETs and benefit from them as a nutrient source (NAD or adenosine) for growth [19]. Thus, future experiments need to verify if neutrophils from stored blood still behave similarly when other pathogens are used for the assays.

In summary, our data show that surviving neutrophils harvested from 24 h stored blood still have comparable or even enhanced antimicrobial activity, as seen in the case of ROS production, phagocytosis or NET formation in response to a selected bacterial pathogen. Nevertheless, it is recommended to analyze exact cell counts and viability of cells, since storage seems to influence the survival of some blood cell populations, including neutrophils. As components of dying cells can be released into the whole blood, it is recommended to critically consider using stored whole blood in ex vivo killing assays. These released components could interfere with bacterial growth and/or killing by neutrophils. However, for all experiments, similar storage times are always recommended during sets of experiments, since storage might slightly impact the antimicrobial efficiency of isolated cells. If shorter storage or other storage conditions (e.g., temperature) do influence the analyzed parameters in a comparable manner, they should be analyzed in future studies.

## 4. Materials and Methods

### 4.1. Collection and Storage of the Blood Samples

The collection of blood from healthy pigs was registered at the Lower Saxonian State Office for Consumer Protection and Food Safety (Niedersächsisches Landesamt für Verbraucherschutz und Lebensmittelsicherheit, No. 33.9-42502-05-18A302). It was conducted in line with the recommendations of the German Society for Laboratory Animal Science (Gesellschaft für Versuchstierkunde) and the German Veterinary Association for the Protection of Animals (Tierärztliche Vereinigung für Tierschutz e. V.) (http://www.gv-solas.de).

The donor pigs were kept in the Clinic for Swine or the Research Center for Emerging Infections and Zoonoses from the University of Veterinary Medicine Hannover, Germany. A total of 30 mL of fresh blood was collected from healthy pigs in S-Monovette^®®^ Lithium-Heparin 9 mL tubes (Sarstedt, Nümbrecht Germany). Half of the blood was analyzed immediately (fresh blood, 0 h) and the rest of the blood was stored in the dark at room temperature for 24 h (stored blood, 24 h). Room temperature was chosen to avoid cold shock of the blood cells.

The same assays were performed with fresh and stored blood for a paired analysis.

### 4.2. Measurement of Free DNA, Histone Fragments and IL17 in Plasma

Plasma samples were collected from the fresh and stored blood. Therefore, 1 mL of the heparinized blood was centrifuged (2100× *g*, 15 min at 20 °C), and the plasma was collected and stored at −20 °C until the respective assays were performed.

A Quant-iT ™ PicoGreen ™ assay (Thermo Fisher, P11496 Invitrogen, Carlsbad, CA, USA) was used, as described previously [35], to determine the amount of free DNA in the plasma samples. The amount of DNA in each sample was calculated based on the standard curve.

The number of histone-associated-DNA-fragments (mono- and oligo-nucleosomes) were quantified with a cell death detection ELISA PLUS kit (Roche Diagnostics GmbH, Mannheim, Germany). The assay was conducted following the manufacturer’s instructions.

The determination of IL17 was analyzed with porcine IL17 ELISA (IL-17 pig ELISA Kit, ab193732 Abcam^®^ Berlin, Germany) following the manufacturer’s instructions.

### 4.3. Blood Smear Analysis

Blood smears were made, air dried and stained with HAEMA quick stain (DIFF Quick, Labor + Technik, Eberhard Lehmann GmbH, Germany, No. LT001), following the manufacturer’s instructions. In the stained smears, the amount of segmented and band neutrophils (in 100 neutrophils) were counted using a light microscope (LEICA DM IL LED, HI Plan I 40×/0.50 PH2 objective) and the percentage in each sample was calculated. Example pictures were made with a Zeiss Axio Imager M2 microscope with a Plan-Apochromat 63×/1.4 Oil DIC ∞/0.17 objective.

### 4.4. Counting of White Blood Cells

White blood cells (WBCs) present in the whole blood tubes (S-Monovette^®®^ EDTA K3/Forensic 9 mL, Sarstedt, Nümbrecht Germany) were determined with the Leuko-TIC^®®^ kit (Bioanalytic GmbH, Umkirch, Germany). The samples were analyzed following the manufacturer’s instructions. The cells were counted in a Neubauer chamber with a light microscope (Leica DM IL LED, HI Plan I 10×/0.25 PH1 objective).

### 4.5. Bacterial Strain and Growth Conditions

*Streptococcus* (*S*.) *suis cps* type 2 strain 10 [32] (NET induction) and *Streptococcus* (*S*.) *suis* 10*cps*Δ*EF* (used in the killing assay as a mutant strain without a capsule that is sensitive to phagocytosis), kindly provided by Hilde Smith (Wageningen, GE, The Netherlands) [32], were used in this study. Therefore, working cryostocks were produced. *S*. *suis* strains were grown on a Columbia blood agar plate (Columbia Agar with 7% Sheep Blood; Thermo Scientific^TM^ PB5008A, Waltham, MA, USA) at 37 °C without CO_2_ (20–24 h). Two colonies were inoculated in 10 mL Todd Hewitt Broth (THB) ((BD Bacto™ Dehydrated Culture Media: Todd Hewitt Broth; Becton Dickinson, 249240, Franklin Lakes, NJ, USA) and incubated at 37 °C for around 16 h in a melting ice-bath to delay start of growth. A 1:50 dilution from the overnight culture was conducted in pre-warmed THB (total 50 mL) and incubated at 37 °C until it reached an optical density of OD_600nm_ ~ 0.85 ± 0.05 (*S*. *suis* 10*cps*Δ*EF*) and OD_600nm_ = 1.15 ± 0.05 (*S. suis cps* type 2 strain 10), the late exponential growth phase. Immediately, the culture was mixed with glycerol (final concentration of 15%) and aliquots in 1.5 mL tubes were shock frosted in liquid nitrogen. The cryostocks were stored at −80 °C until they were used and only thawed once.

### 4.6. Whole Blood Killing Assay

A total of 500 μL blood was mixed in a 1.5 mL tube with *S. suis* 10*cps*Δ*EF* (high infection dose = 1.5 × 10^6^ CFU/mL or low infection dose = 1.5 × 10^5^ CFU/mL). Samples were incubated with and without 10 μg/mL (final concentration) of cytochalasin D (Sigma-Aldrich, Munich, Germany C2618), a fungal toxin that inhibits phagocytosis. Samples were incubated for 2 h on a rotator (7 rpm) at 37 °C. To determine the CFU/mL, serious dilutions at the time points 0 and 2 h were plated on Columbia blood agar plates (Columbia Agar with 7% Sheep Blood; Thermo Scientific^TM^ PB5008A, Waltham, MA, USA) and incubated for 20–24 h at 37 °C. The survival factor (SF) was calculated with the formula SF_2h_ = CFU_2h_/CFU_0h_ [44].

### 4.7. Neutrophil Isolation

Porcine neutrophils were purified from the whole blood using a density gradient with Biocoll (1.077 g/mL, Biochrom, L 6115, Berlin, Germany) as previously described [19,35]. The final cell pellet was resuspended in 1 mL cold Roswell Park Memorial Institute (RPMI) 1640 Medium, without phenol red (Thermo Fisher, 11835063 Gibco™ RPMI 1640 Medium, no phenol red, Carlsbad, CA, USA). The cells were stained with trypan blue (1680.1 Carl Roth^®®^, Karlsruhe, Germany) and counted in a Neubauer chamber. The cell number was adjusted with RPMI to the needed cell concentration depending on the experiment.

### 4.8. Analysis of Isolated Neutrophils (Viability and ROS Production)

Purified neutrophils were analyzed for purity, granularity, viability and ROS production by flow cytometry analysis using Attune^®®^ NxT Acoustic Focusing Flow Cytometer, Invitrogen (Laser 488 nm (50 mW), filter BL1 = 530/30 (ROS), BL2 = 590/40 (PI)). The acquisition volume was set to 100 μL, the acquisition speed was set to 100 μL/min and a total of 10,000 events were recorded. Cells were adjusted to 5 × 10^5^ cells/mL in 200 μL.

The purity of neutrophils was analyzed after exclusion of debris in all viable cells based on FSC-A and SSC-A settings. Example picture’s for the gating strategy are presented in Figure 3d,e.

The number of dead cells in all isolated cells was detected with propidium iodide (PI) staining (1.0 mg/mL in water, P4864 Sigma-Aldrich, Munich, Germany) following the manufacturer’s instructions. A dead control (10 min, 70 °C heat block) and a 1:2 diluted dead:living cell control was used to adjust the flow cytometer settings. The gating strategy is presented in Figure 3c.

To determine ROS production in isolated neutrophils, cells were incubated with and without phorbol 12-myristate 13-acetate (PMA, 25 nM final concentration, 524,400 Sigma Aldrich, Munich, Germany). Intracellular ROS production was measured by immediately adding 2′,7′- dichlorofluorosceindiacetate (DCFH-DA, 10 μM) to each sample. All samples were incubated at 37 °C and 5% CO_2_ for 30 min. Respective background controls (without DCFH-DA) were included in all assays. The mean green fluorescence intensity of all cells (X-Mean of BL-1) was recorded by flow cytometer as a relative measure of ROS production. The gating strategy excluded debris and gates were set to the neutrophil population. Data were analyzed with FlowJo^TM^10.6.1 software (Ashland, OR, USA).

### 4.9. Neutrophil Killing Assay

In each well of 48 well plates (Greiner Bio-One, 677102, Kremsmünster, Austria), 2 × 10^5^/100 μL porcine neutrophils were seeded and infected with *S. suis* 10*cps*Δ*EF* (MOI = 1). Samples were incubated with and without 10% of autologous porcine plasma and 10 μg/mL (final concentration) of cytochalasin D (Sigma-Aldrich, Munich, Germany C2618). RPMI medium was added to complete the volume of each well to 190 μL. The plates were centrifuged (370× *g*, 5 min) and were incubated for 2 h at 37 °C, 5% CO_2_. The survival factor (SF) was determined as described in 4.6.

### 4.10. Transmigration of Neutrophils from Whole Blood

In each well of 24 well plates (Greiner Bio-One, Kremsmünster, Austria), 1 mL RPMI was added with and without IL-8 (recombinant porcine IL-8/CXCL8 protein (535-IN-025 R&D Systems, Bio-Techne GmbH, MN, USA), final concentration 100 ng/mL). In each well, a sterile transwell filter with 3 μm pore size (thincert cell culture insert 662631, Greiner Bio-One, Kremsmünster, Austria) was added. Inside the transwell, 400 μL whole blood was added and the plate was incubated for 4 h at 37 °C, 5%CO_2_. At the end of the incubation, the transwell filters were taken out from the wells. Each well was mixed 10 times by pipetting up and down 1 mL. From each well, 500 μL was fixed with paraformaldehyde (4% final concentration). The fixed cells were analyzed based on forward (FSC) and sideward (SSC) scatter using Attune^®®^ NxT Acoustic Focusing Flow Cytometer (Invitrogen, Carlsbad, CA, USA). Each sample was conducted in triplicates per individual run. Data were analyzed with FlowJo^TM^10.6.1 software (Ashland, OR, USA) by drawing a gate to exclude the debris and on the individual cell type populations based on FSC-A and SSC-A, as presented in Figure 4b.

### 4.11. NET Induction in Isolated Neutrophils

Cover slips (8 mm; Thermo Fisher Scientific (Bremen) GmbH) for the NET induction were used in 48 well plates and they were coated in accordance with the manufacturer’s instructions with poly-L-lysine (0.01% solution P4707, Sigma Aldrich, Munich, Germany) and handled afterwards as previously described [19]. In each well, 2 × 10^5^ neutrophils/100 μL were seeded. As a negative control, RPMI medium was added. As a positive control, 100 μL methyl-β-cyclodextrin (10 mM final concentration, C4555 Sigma Aldrich, Munich, Germany) or 100 μL phorbol 12-myristate 13-acetate (PMA, 25 nM final concentration, 524,400 Sigma Aldrich, Munich, Germany) was added. The neutrophils were infected with *S. suis cps* type 2 strain 10 (MOI = 2). The plates were centrifuged (370× *g*, 5 min) and incubated at 37 °C, 5% CO_2_. After 3 h of incubation, the samples were fixed with paraformaldehyde (4% final concentration) and the plates were wrapped with parafilm and stored at 4 °C until the staining was conducted.

### 4.12. NET Staining

NETs were stained as previously described [45]. After the permeabilization and the blocking of the samples, these were incubated with the first antibodies: a mouse monoclonal antibody (IgG2a) against DNA/histone 1 (MAB3864; Millipore 0.55 mg/mL diluted 1:1000, Billerica, MA, USA) and a rabbit anti-human myeloperoxidase (A039829-2 Agilent, Santa Clara, CA, USA, 3.2 mg, 1:337.5), for 1 h at room temperature. For the isotype controls murine IgG2a (from murine myeloma, M5409-0.2 mg/mL, 1:364 Sigma Aldrich, Munich, Germany) and rabbit IgG (from rabbit serum, Sigma Aldrich, Munich, Germany, I5006, 1.16 mg, 1:10,875) were used. As secondary antibodies, goat anti-mouse Alexa 488Plus (1:500, Invitrogen, Carlsbad, CA, USA) and goat anti-rabbit Alexa 633 (Thermo Scientific, 2 mg, 1:500, Waltham, MA, USA) were used and incubated for 1 h at room temperature in the dark. Afterwards, the samples were washed three times with 1× PBS (phosphate buffered saline) and one time with distilled water. The samples were stained 10 min (in the dark, room temperature) with aqueous Hoechst 33,342 (1:1000, stock 50 mg/mL, Sigma Aldrich, Munich, Germany). The slides were then washed three times with distilled water and embedded in 3–5 μL ProlongGold (without DAPI, Invitrogen, Carlsbad, CA, USA). The samples were dried over night at 4 °C and all cover slips were surrounded with clear nail polish. Samples were stored at 4 °C in the dark until analysis.

### 4.13. NET Quantification

For each sample, six pictures were made randomly. The pictures were taken by a Leica TCS SP5 AOBS confocal inverted-base fluorescence microscope with an HCX PL APO 40× 0.75–1.25 oil immersion objective. The cells present in the pictures were counted manually using ImageJ software (version 1.52q, National Institute of Health, USA). The total number of neutrophils and positive neutrophils (activated or NET-releasing) were counted. A neutrophil was counted as positive if an evident off-shoot of DNA was visible or if at least two of the following criteria were found: enlarged nucleus, decondensed nucleus or blurry rim. The percentage of NET-positive neutrophils was calculated. An average from the six pictures form each sample was made.

### 4.14. Cholesterol Analysis

In 1.5 mL tubes, 3 × 10^6^ isolated neutrophils in 100 μL RPMI were added and stimulated with methyl-ß-cyclodextrin (10 mM final concentration, C4555 Sigma Aldrich, Munich, Germany) or not stimulated. For control, RPMI medium was used. The final volume was 200 μL. The samples were incubated for 180 min at 37 °C, 5% CO_2_ without closing the lid completely. After the incubation, the samples were centrifuged (400× *g*, 5 min). The supernatant was discarded carefully by pipetting. The pellet was washed twice with 500 μL of 1× PBS (Lipopolysaccharides (LPS) free) and centrifuged (400× *g*, 5 min). The final pellet was resuspended in 500 μL pure SIGMA water (for RNA/DNA work) and the tubes were stored at −20 °C until the cholesterol analysis. The lipid isolation and cholesterol and oxysterol analysis was performed as previously described [41,46].

### 4.15. Statistical Analysis

Data were analyzed using Excel 2016 or 2018 (Microsoft) and GraphPad Prism 8.3 or 8.4.1(460) (GraphPad Software). Data were analyzed with one-tailed paired or unpaired Student’s *t*-test and presented with mean ± SD and the differences between groups were analyzed as described in the figure legends (* *p <* 0.05, ** *p <* 0.01, *** *p <* 0.001, **** *p <* 0.0001).

## 5. Conclusions

We conclude that the storage of blood slightly affects the viability of neutrophils, as we isolated slightly less neutrophils from stored blood. However, the surviving neutrophils from stored blood did show comparable morphology and/or even enhanced antimicrobial activity, as shown by phagocytosis of *S. suis*, NET formation, ROS production and transmigration in response to IL8. Thus, even if they are isolated in less quantity, neutrophils isolated from stored blood could still be used for certain assays since they still exhibit antimicrobial activity. However, the differences that could occur depending on the stimulus or the pathogens used for the infection studies with neutrophils must be considered. In particular, the storage conditions should always stay constant during one set of experiment to prevent negative influence on the results and higher variances that may occur after varying blood storage times.
